# How Can Manufacturers Promote Green Innovation in Food Supply Chain? Cost Sharing Strategy for Supplier Motivation

**DOI:** 10.3389/fpsyg.2020.574832

**Published:** 2020-09-18

**Authors:** Jianhong He, Yaling Lei, Xiao Fu, Chia-Hsun Lin, Chih-Hsiang Chang

**Affiliations:** ^1^School of Economics and Management, Chongqing University of Posts and Telecommunications, Chongqing, China; ^2^Department of Leisure and Sport Management, Cheng Shiu University, Kaohsiung, China

**Keywords:** food supply chain, cost sharing, supply interruption, motivation, green innovation

## Abstract

In the innovation of production activities by green product manufacturing or application, food supply chain cooperation is an important method to optimize the allocation of internal and external innovation resources, strengthen their own core capabilities and achieve sustainable development of enterprises. Whether the traditional revenue sharing or cost sharing strategy is still efficient in the food supply chain cooperation aiming at green innovation attracts a lot of attention. Further research about whether the traditional cooperation contract can effectively motivate suppliers to maximize their innovation efforts is required. In this paper, the green innovation effort level parameters are designed and the constraint factor of the green preference of consumers at the market end is applied to discuss the incentive strategy of cost sharing led by manufacturers. Stackelberg equilibrium structure is utilized in the incentive model in this paper to discuss the existence of the optimal cost sharing ratio, the optimal effort level and the optimal income of green innovation cooperation in the food supply chain. The results show that when the supply is interrupted due to the insufficient stimulation of green consumption at the market demand side, manufacturers need to stimulate their green innovation efforts by sharing the cost of suppliers, and the cost sharing proportion is affected by the marginal profit coefficient of manufacturers and suppliers. When the relationship between the marginal profit of suppliers and the marginal profit of manufacturers reaches a certain threshold, manufacturers use the cost sharing contract, which can effectively stimulate the green innovation efforts of suppliers and optimize the overall income of the food supply chain.

## Introduction

In the innovation of production activities by green product manufacturing or application, supply chain cooperation is an important means to optimize the allocation of internal and external innovation resources, strengthen their own core capabilities, and achieve sustainable development of enterprises (Shah et al., 2018). In the complex external environment, how to coordinate the relationship between member enterprises is the basic problem of supply chain management, and the establishment of a cooperation mechanism that can motivate all parties is the focus of enterprise relationship in the food supply chain (Sunny and Shu, 2019). This is particularly important for the supply chain green innovation activities with long R&D cycle and high complexity, due to the high uncertainty of the results of innovation activities, the information asymmetry characteristics in innovation cooperation, and the dilution effect of Technology Spillover on the innovation income of specific enterprises (Iyer and Soberman, 2016). It is difficult for enterprises to obtain the favor of capital market in the initial stage of green innovation activities, and the decision-making process of green innovation activities is long. These make the close strategic cooperation and coordination between the upstream and downstream enterprises in the food supply chain extremely important. Traditionally, the cooperation mode between upstream and downstream enterprises in the food supply chain includes two ways: one is to invest in the other party and provide guarantee for the other party's income to encourage it to join the cooperation, such as short-term capital injection, joint venture, integrated operation and other single capital behaviors (Gui et al., 2018); the other is to complement resources and share risks through long-term contracts, strategic cooperation and other ways, such as through revenue benefit sharing contract, wholesale price premium contract, cost sharing contract, and other ways to achieve diversified cooperation (Ghosh and Shah, 2015; Mensah et al., 2019; Post et al., 2019). These collaborations are usually based on a clear understanding of demanding a stable market and knowing the cost-benefit status of each cooperation subject, with the goal of maximizing the current profit of the food supply chain as the decision-making goal, and focus on discussing what kind of contractual or non- contractual form will bring the best profit to all participants to reach an optimal decision.

Compared with the traditional food supply chain cooperation incentive, the green innovation activities in the food supply chain need to take into account both economic benefits and environmental performance. In the cooperation mode, more attention is paid to the effort level and long-term strategic cooperation willingness of food supply chain members. The effort level of members determines the quality and efficiency of the green innovation activities in the supply chain (Truong and Berrone, 2015; Hafezalkotob, 2017). Current research shows that manufacturers are more likely to become the initiators and leaders of green innovation activities, and seek guidance or control over upstream suppliers, because they are closer to the market and more sensitive to the information contained in the market such as consumers' green preferences and price effects (Zissis et al., 2015). They can make use of the advantages of leading force and asymmetric information in product design or technical scheme formulation, which enable them to maximize personal benefits. They can also use cost subsidies, revenue sharing and other ways to stimulate the innovation efforts of suppliers to maximize system revenue (Lou et al., 2018; Hong and Guo, 2019). Apart from that, there have been many meaningful researches on introducing cost sharing into food supply chain strategic cooperation or innovation cooperation. For example, some research have explored the existence of the relationship among sharing coefficient, unit revenue and effort degree in food supply chain cost sharing, and whether the relationship is affected by game elements such as decision-making environment, decision-making order and decision-making purpose (Liao et al., 2018; Jung et al., 2019; Valero et al., 2019); There are also discussion about the effects of different cost sharing objects, sharing quantity and sharing periodicity on cost sharing strategy selection (Kunapatarawong and Martinez-Ros, 2014; Geng and Dai, 2018).

The cost sharing ratio of manufacturers and suppliers may have a more profound impact on the depth of cooperation and the probability of successful cooperation between the two sides with the guidance of green innovation. It also plays a decisive role in stimulating the efforts of suppliers, which worth more attention (Yalabik and Fairchild, 2011; Parker and Van Alstyne, 2017; Gui et al., 2018). Also, when the manufacturer is assumed to be the initiator and leader of green innovation cooperation, the innovation efforts (Agrawal and Ülkü, 2012; Hong and Guo, 2019), net profit level (Töbelmann and Wendler, 2020), cooperation scale (Yenipazarli, 2017; Aragon-Correa et al., 2018) and R&D cost (Madani and Rasti-Barzoki, 2017) in the supply chain cooperation may change, especially when the non-linear change of cost sharing ratio may bring new impact on the green innovation decision-making of the food supply chain. This is especially when the manufacturer seeks optimal solution of green innovation incentives (Chen et al., 2019; Abbas, 2020). Based on this, the impact of the change of cost sharing coefficient on the green innovation decision-making of the whole food supply chain in the manufacturer led food supply chain green innovation cooperation will be explored in this paper. It will also be discussed how the manufacturer can effectively motivate the supplier through cost sharing, so as to improve the level of green innovation efforts, realize Pareto improvement of the food supply chain system profits, and promote the whole food supply chain green innovation activities with sustainable growth and green development.

## Literature Review Problem Description and Symbol Description

### Problem Description

In this paper, a secondary food supply chain composed of upstream intermediate product supplier (s) and downstream final product manufacturer (m) is studied. In the face of the green demand of consumers in the market, manufacturers have a higher willingness to innovate. So it is assumed in this paper that manufacturers are the initiators of green innovation cooperation. For example, in the food supply chain, when there is an increasing green preference of consumers, the manufacturers encourage suppliers to jointly improve the energy exhaust system, improve the fuel utilization efficiency or adopt green innovation activities such as new energy and new power system to provide the market with higher green degree products, so as to obtain a higher market share. In the traditional supplier manufacturer relationship, these incremental revenues are mainly obtained by the manufacturer, and the supplier can only obtain certain indirect income through the increase of supply. Therefore, the incentive in the incremental sales of green products is very limited, which is prone to supply interruption (Cohen et al., 2015). At the same time, due to the high cost of green innovation and the high risk and uncertainty in the innovation process (Bendell, 2016), in order to effectively control the investment scale and reduce the investment risk, manufacturers often hope that suppliers can make more green innovation efforts, so they take measures to provide suppliers with a certain share of green innovation cost, so as to encourage suppliers' enthusiasm for green innovation and promote suppliers to participate in green innovation in depth New activities to realize resource coordination of green innovation in food supply chain.

### Symbol Description

In this section, the main parameters and variable symbols involved in the later game model construction and reasoning are described according to the definition specification of food supply chain in Table 1. Some parameters and variables only appear in individual models, which are not listed in this table. These will be explained when they appear.

**Table 1:** Symbol description.

**Symbol**	**Description**
*I*_*i*_(*t*)	The level of green innovation efforts of food supply chain members at a specific time
*C*_*i*_	Cost of green innovation efforts of food supply chain members
*k*_*i*_	Green innovation cost coefficient of food supply chain members
φ	Manufacturers' share of green innovation cost to suppliers
*J*_*i*_	Net present value of food supply chain members' profits at a specific time
$\prod_{i}^{j}$	Profit of food supply chain members in the context of cost sharing
ρ_*i*_	Marginal profit of products of member units in food supply chain
ρ	Discount rate
*j* = *d*	Stackelberg game under cost sharing
*i* = {*m, s*}	Supply chain members (manufacturers, suppliers)

## The Game Model of Manufacturer Providing Cost Sharing

### Model Assumptions

(1) All members of the food supply chain are rational subjects, which means manufacturers will maximize their profit and consumers will pursue optimal utility. The cost of green innovation activities is affected by their own level of green innovation efforts (Bray et al., 2019), which is continuously rising with the increase of the level of green innovation efforts. Considering the convex characteristics of the cost of green innovation activities, it is assumed that the cost of green innovation of members of the food supply chain is *I*_*i*_(*t*), and a convex function $C_{i}=\frac{1}{2}k_{i}I_{i}^{2}\left(t\right)$, (*i* = *m, s*). And since green innovation belongs to one-off scientific research investment, it will not affect the fixed production cost of unit product. The higher the level of green innovation efforts, the green degree of the product will be higher, and more environmentally friendly. In practice, enterprises transmit their green innovation input and other information to consumers through ecological labels.

(2) Same with Stefano (Ramanathan et al., 2018), the reference price model which combines memory and stimulation is adopted in this paper adopts. And it is assumed that the change of reference price of products follows the following dynamic equation:

$$\begin{array}{l} \overset{•}{r\left(t\right)}=\alpha \left[p-r\left(t\right)\right]+\beta \left[I_{m}\left(t\right)+I_{s}\left(t\right)\right]\\ r\left(0\right)=r0 \end{array}$$

Where *r*(*t*) is the reference price of the product at the moment *t*, $\overset{•}{r\left(t\right)}=\frac{dr\left(t\right)}{dt}$ is the change rate of the reference price *r*(*t*) at the moment *r*(*t*), *r*_0_ is the initial reference price of the product *i*. α(α > 0) is the “memory parameter” of the consumer, and β(β > 0) represents the influence factor of the green innovation input of the product on the reference price.

(3) It is assumed that the green attribute of the product can stimulate the market demand, and the demand is a linear function of the level of green innovation efforts (Ramanathan et al., 2018). On the basis of classical function, the influence of consumer's green preference behavior and reference price effect on market demand is introduced as below:

$$\begin{array}{l} Q\left(t\right)=a-bp+\delta \left[r\left(t\right)-p\right]+\eta \left[I_{m}\left(t\right)+I_{s}\left(t\right)\right] \end{array}$$

It can be seen from the above functions that the actual demand of consumers for green products is affected by the sales price, green degree level and reference price effect of green products. *a*(*a* > 0) refers to the potential market demand of green products, *b*(*b* ∈ (0, 1)) refers to the price elasticity coefficient of demand *Q*. δ(δ > 0) refers to the reference price coefficient, which shows the sensitivity between the actual price and the reference price of consumers, that also means the reference price effect. In special cases, δ = 0 refers to no reference price effect. In addition, η(η ∈ [0, 1]) indicates the sensitivity coefficient of consumers' green degree. The larger the coefficient η is, the more consumers prefer green products.

### Model Establishment and Solution

In the supply chain green innovation cooperation with cost sharing as the link, manufacturers play a leading role while suppliers play a follower role. In order to encourage suppliers to participate in green innovation activities, manufacturers provide suppliers with a certain proportion of cost sharing. From the perspective of long-term and dynamic equilibrium, the two decisions on the level of green innovation efforts constitute a Stackelberg differential game model between upstream and downstream manufacturers.

Manufacturers and suppliers make independent decisions to maximize their profits. In the first stage, manufacturers decide their own level *I*_*m*_(*t*) of green innovation efforts and the proportion φ(*t*) of green innovation cost sharing provided to suppliers. In the second stage, suppliers decide their own level *I*_*s*_(*t*) of green innovation efforts according to the given *I*_*m*_(*t*) and φ(*t*). The manufacturer's decision-making problem can be obtained via below:

(1) $$\begin{array}{r} \underset{I_{m},\varphi }{\max}J_{m}^{d}=\int_{0}^{\infty }e^{-\rho t}\left\{\rho_{m}Q-C_{m}\left(I_{m}\right)-\varphi C_{s}\left(I_{s}\right)\right\}dt \end{array}$$

Given *I*_*m*_ and φ, the decision problem of the supplier is:

(2) $$\begin{array}{r} \underset{I_{s}}{\max}J_{s}^{d}=\int_{0}^{\infty }e^{-\rho t}\left\{\rho_{s}Q-\left(1-\varphi \right)C_{s}\left(I_{s}\right)\right\}dt \end{array}$$

**Proposition 1:** The optimal level of green innovation effort of manufacturer is $I_{m}^{d^{*}}=\frac{\rho_{m}\left(\rho \eta +\alpha \eta +\beta \delta \right)}{k_{m}\left(\rho +\alpha \right)}$. The optimal level of green innovation effort of supplier is $I_{s}^{d^{*}}=\frac{\left(2\rho_{m}+\rho_{s}\right)\left(\rho \eta +\alpha \eta +\beta \delta \right)}{{2k}_{s}\left(\rho +\alpha \right)}$. The optimal cost sharing ratio of manufacturer to supplier is $\varphi^{d^{*}}=\frac{2\rho_{m}-\rho_{s}}{2\rho_{m}+\rho_{s}}$. And the optimal profit value function of manufacturer and supplier is

$$\begin{array}{l} \{\begin{array}{c} J_{m}^{d^{*}}\left(r,t\right)=e^{-\rho t}\left(a_{1}^{d^{*}}r+b_{1}^{d^{*}}\right)\\ J_{s}^{d^{*}}\left(r,t\right)=e^{-\rho t}\left(a_{2}^{d^{*}}r+b_{2}^{d^{*}}\right). \end{array} \end{array}$$

$$\begin{array}{l} \{\begin{array}{l} a_{1}^{d^{*}}=\frac{\rho_{m}\delta }{\rho +\alpha }\\ a_{2}^{d^{*}}=\frac{\rho_{s}\delta }{\rho +\alpha }\\ b_{1}^{d^{*}}=\frac{\rho_{m}}{\rho }\left[\alpha -bp-\delta p+\eta \left(\frac{\rho_{m}\eta +\beta a_{1}^{d}}{k_{m}}+\frac{{2\rho_{m}\eta +\rho }_{s}\eta +2\beta a_{1}^{d}+\beta a_{2}^{d}}{{2k}_{s}}\right)\right]\\ -\frac{{\left(\rho_{m}\eta +\beta a_{1}^{d}\right)}^{2}}{{2\rho k}_{m}}-\frac{{\left({2\rho }_{m}\eta +2\beta a_{1}^{d}\right)}^{2}-{\left(\rho_{s}\eta +\beta a_{2}^{d}\right)}^{2}}{{8\rho k}_{s}}\\ +\frac{a_{1}^{d}}{\rho }\left[\begin{array}{l} \alpha p+\\ \beta \left(\frac{\rho_{m}\eta +\beta a_{1}^{d}}{k_{m}}+\frac{{2\rho_{m}\eta +\rho }_{s}\eta +2\beta a_{1}^{d}+\beta a_{2}^{d}}{{2k}_{s}}\right) \end{array}\right]\\ b_{2}^{d^{*}}=\frac{\rho_{s}}{\rho }\left[\alpha -bp-\delta p+\eta \left(\frac{\rho_{m}\eta +\beta a_{1}^{d}}{k_{m}}+\frac{{2\rho_{m}\eta +\rho }_{s}\eta +2\beta a_{1}^{d}+\beta a_{2}^{d}}{{2k}_{s}}\right)\right]\\ -\frac{{\left({2\rho }_{m}\eta +\rho_{s}\eta +2\beta a_{1}^{d}+\beta a_{2}^{d}\right)}^{2}}{{8\rho k}_{s}}\\ +\frac{{\left({2\rho }_{m}\eta +2\beta a_{1}^{d}\right)}^{2}-{\left(\rho_{s}\eta +\beta a_{2}^{d}\right)}^{2}}{{8\rho k}_{s}}+\frac{a_{1}^{d}}{\rho }\left[\begin{array}{l} \alpha p+\\ \beta \left(\frac{\rho_{m}\eta +\beta a_{1}^{d}}{k_{m}}+\frac{{2\rho_{m}\eta +\rho }_{s}\eta +2\beta a_{1}^{d}+\beta a_{2}^{d}}{{2k}_{s}}\right) \end{array}\right] \end{array} \end{array}$$

**Prove:** The optimal profit function of the supplier at time t can be expressed as:

(3) $$\begin{array}{r} J_{s}^{d^{*}}\left(r,t\right)=e^{-\rho t}V_{s}^{d}\left(r\right) \end{array}$$

while, $V_{s}^{d}\left(r\right)=\underset{I_{s}}{\max}\int_{t}^{\infty }e^{-\rho \left(s-t\right)}\left\{\rho_{s}Q-\left(1-\varphi \right)C_{s}\left(I_{s}\right)\right\}ds$.

It can be seen that the supplier optimal control problem satisfies the following HJB equation:

(4) $$\begin{array}{r} \rho V_{s}^{d}\left(r\right)=\underset{I_{s}}{\max}\{\rho_{s}\left[a-bp+\delta \left(r-p\right)+\eta \left(I_{m}+I_{s}\right)\right]\\ -\frac{1}{2}\left(1-\varphi \right)k_{s}I_{s}^{2}+V_{s}^{d^{\prime }}\left(r\right)\left[\alpha \left(p-r\right)+\beta \left(I_{m}+I_{s}\right)\right]\} \end{array}$$

Formula (4) is known to be a concave function of *I*_*s*_. The following equation can be obtained according to the first order condition:

(5) $$\begin{array}{r} I_{s}=\frac{\rho_{s}\eta +\beta V_{s}^{d^{\prime }}\left(r\right)}{\left(1-\varphi \right)k_{s}} \end{array}$$

In the same way, the optimal profit function of manufacturer at time t can be expressed as:

(6) $$\begin{array}{r} J_{m}^{d^{*}}\left(r,t\right)=e^{-\rho t}V_{m}^{d}\left(r\right) \end{array}$$

within this, $V_{m}^{d}\left(r\right)=\underset{I_{m},\varphi }{\max}\int_{t}^{\infty }e^{-\rho \left(s-t\right)}\left\{\rho_{m}Q-C_{m}\left(I_{m}\right)-\varphi C_{s}\left(I_{s}\right)\right\}ds$.

It can be seen that the manufacturer's optimal control problem satisfies the following HJB equation:

(7) $$\begin{array}{r} \rho V_{m}^{d}\left(r\right)=\underset{I_{m},\varphi }{\max}\left\{\rho_{m}Q-C_{m}-\varphi C_{s}+V_{m}^{n^{\prime }}\left(r\right)\overset{\cdot }{r}\right\} \end{array}$$

Take the response function (5) of the supplier into Equation (7) and expand to obtain the following:

(8) $$\begin{array}{r} \rho V_{m}^{d}\left(r\right)=\underset{I_{m},\varphi }{\max}\{\rho_{m}\left[a-bp+\delta \left(r-p\right)+\eta \left(I_{m}+\frac{\rho_{s}\eta +\beta V_{s}^{d^{\prime }}\left(r\right)}{\left(1-\varphi \right)k_{s}}\right)\right]\\ -\frac{1}{2}k_{m}I_{m}^{2}\\ -\frac{\varphi }{2}k_{s}{\left[\frac{\rho_{s}\eta +\beta V_{s}^{d^{\prime }}\left(r\right)}{\left(1-\varphi \right)k_{s}}\right]}^{2}+V_{m}^{d^{\prime }}\left(r\right)[\alpha \left(p-r\right)\\ +\beta \left(I_{m}+\frac{\rho_{s}\eta +\beta V_{s}^{d^{\prime }}\left(r\right)}{\left(1-\varphi \right)k_{s}}\right)]\} \end{array}$$

According to the Haisai matrix, formula (8) is a concave function with respect to *I*_*m*_ and φ, which can be obtained from the first order conditional formula as below:

(9) $$\begin{array}{r} I_{m}=\frac{\rho_{m}\eta +\beta V_{m}^{d^{\prime }}\left(r\right)}{k_{m}},\;\varphi =\frac{2\rho_{m}\eta -\rho_{s}\eta +2\beta V_{m}^{d^{\prime }}\left(r\right)-\beta V_{s}^{d^{\prime }}\left(r\right)}{2\rho_{m}\eta +\rho_{s}\eta +2\beta V_{m}^{d^{\prime }}\left(r\right)+\beta V_{s}^{d^{\prime }}\left(r\right)} \end{array}$$

By introducing *I*_*m*_, *I*_*s*_ and φ into Equations (4) and (8), we can infer that the linear optimal value function of r is the solution of HJB equation.

So the expression of function sum $V_{m}^{d}\left(r\right)$ and $V_{s}^{d}\left(r\right)$ can be expressed as:

(10) $$\begin{array}{r} V_{m}^{d}\left(r\right)=a_{1}^{d}r+b_{1}^{d},\;V_{s}^{d}\left(r\right)=a_{2}^{d}r+b_{2}^{d} \end{array}$$

Within this equation, $a_{1}^{d}$, $b_{1}^{d}$, $a_{2}^{d}$, $b_{2}^{d}$ are all unknown constants. If equation (10) is taken into the arrangement, the constraint equations can be obtained about $a_{1}^{d}$, $b_{1}^{d}$, $a_{2}^{d}$, $b_{2}^{d}$. Solve the equations, $a_{1}^{d}$, $b_{1}^{d}$, $a_{2}^{d}$, $b_{2}^{d}$ can be obtained. Bring it into equation (10), the expression of function $V_{m}^{d}\left(r\right)$ 和 $V_{s}^{d}\left(r\right)$ can be obtained:

(11) $$\begin{array}{r} V_{m}^{d}\left(r\right)=a_{1}^{d^{*}}r+b_{1}^{d^{*}},\;V_{s}^{d}\left(r\right)=a_{2}^{d^{*}}r+b_{2}^{d^{*}} \end{array}$$

By substituting Equation (11) and its first derivative into Equations (5) and (9), we can see that the equilibrium solutions of manufacturers and suppliers are, respectively, below.

$$\begin{array}{r} I_{m}^{d^{*}}=\frac{\rho_{m}\left(\eta \rho +\eta \alpha +\delta \beta \right)}{k_{m}\left(\rho +\alpha \right)},\;\varphi^{d^{*}}=\frac{2\rho_{m}-\rho_{s}}{2\rho_{m}+\rho_{s}},I_{s}^{d^{*}} \end{array}$$

(12) $$\begin{array}{rc} = & \frac{\left(2\rho_{m}+\rho_{s}\right)\left(\eta \rho +\eta \alpha +\delta \beta \right)}{2k_{s}\left(\rho +\alpha \right)} \end{array}$$

Taking Equation (12) into Equation (1) and Equation (4), it can be get the optimal profit function of manufacturers and suppliers.

**Proposition 2:** At that time $-2<\frac{\rho_{s}}{\rho_{m}}\leq 0$, the optimal level of green innovation efforts of manufacturers and suppliers was positive. The green innovation activities of the whole food supply chain were in the “double driving” mode, which means they were continuously influenced by the green preferences of consumers at the demand end of the external market, the reference price effect and the positive incentive of internal cost sharing, in which the incentive from internal cost sharing was the secondary part.

$$\begin{array}{l} \text{Proof}:\text{ Because }I_{m}^{d^{*}}=\frac{\rho_{m}\left(\rho \eta +\alpha \eta +\beta \delta \right)}{k_{m}\left(\rho +\alpha \right)},\\ I_{s}^{d^{*}}=\frac{\left(2\rho_{m}+\rho_{s}\right)\left(\rho \eta +\alpha \eta +\beta \delta \right)}{{2k}_{s}\left(\rho +\alpha \right)},\text{ we can get} \end{array}$$

$$\begin{array}{l} \{\begin{array}{l} \rho_{m}\leq 0,I_{m}^{d^{*}}\leq 0\\ \rho_{m}>0,I_{m}^{d^{*}}>0\\ \frac{\rho_{s}}{\rho_{m}}>-2,I_{s}^{d^{*}}>0 \end{array} \end{array}$$

At that time $-2<\frac{\rho_{s}}{\rho_{m}}\leq 0$, the optimal level of green innovation efforts of manufacturers and suppliers was positive.

It is shown with further analysis of Proposition 2 that, at that time ρ_*m*_ ≤ 0, the manufacturer's optimal green innovation effort level is negative or zero, which means they should cut down investment on innovation. At this time, the manufacturer will not choose to carry out green innovation activities. At that time ρ_*m*_ > 0, the manufacturer's optimal green innovation effort level is positive, which indicate that at this time, the consumer's green preference and reference price effect have obviously encouraged the manufacturer's green innovation willingness. At that time 2ρ_*m*_ + ρ_*s*_ > 0, the supply. The level of green innovation efforts is positive, which shows that the demand for green products at the market demand side and the cost sharing of manufacturers to suppliers can play an effective incentive role. To sum up, at that time $-2<\frac{\rho_{s}}{\rho_{m}}\leq 0$, the green innovation efforts of suppliers and manufacturers were positively encouraged, and the green innovation activities of the whole supply chain were in the “double driving” mode. This means that they were continuously stimulated by the incentives of green preferences of consumers in the demand side of the external market and the reference price effect and the internal cost sharing.

**Corollary 1:** At that time $\frac{\rho_{s}}{\rho_{m}}\leq -2$, the level of green innovation efforts of suppliers was negative. For suppliers, the marginal profit of green raw materials was too low, and there was basically no profit space. Providing green raw materials to manufacturers could not help suppliers to achieve the annual profit goals of enterprises, and the suppliers' subjective willingness to carry out green innovation activities was low, so there would be supply interruption. At the same time, the demand stimulation from the market end is weak, which cannot directly stimulate the efforts of green innovation activities in the food supply chain. Manufacturers must effectively encourage suppliers through cost sharing, which is greater than normal. Otherwise, the problem of supply interruption will be infinitely enlarged to the whole supply chain through “bullwhip effect,” and even cause the green production in the food supply chain Production and manufacturing of products, logistics, transportation and other aspects of the problem.

**Proposition 3:** There is a threshold $\frac{\rho_{s}}{\rho_{m}}=2$ of marginal profit ratio between suppliers and manufacturers. Within the threshold range $0<\frac{\rho_{s}}{\rho_{m}}\leq 2$, cost sharing has an obvious incentive effect on suppliers' green innovation efforts, and the incentive effect of cost sharing is stronger than that of market demand.

**Proof**: $I_{m}^{d^{*}}-I_{m}^{n^{*}}=\frac{\rho_{m}\left(\eta \rho +\eta \alpha +\delta \beta \right)}{k_{m}\left(\rho +\alpha \right)}-\frac{\rho_{m}\left(\eta \rho +\eta \alpha +\delta \beta \right)}{k_{m}\left(\rho +\alpha \right)}=0$

$$\begin{array}{l} I_{s}^{d^{*}}-I_{s}^{n^{*}}=\frac{\left(2\rho_{m}+\rho_{s}\right)\left(\eta \rho +\eta \alpha +\delta \beta \right)}{{2k}_{s}\left(\rho +\alpha \right)}-\frac{\rho_{s}\left(\eta \rho +\eta \alpha +\delta \beta \right)}{k_{s}\left(\rho +\alpha \right)}\\ =\frac{\left(2\rho_{m}-\rho_{s}\right)\left(\eta \rho +\eta \alpha +\delta \beta \right)}{{2k}_{s}\left(\rho +\alpha \right)}\\ 当\rho_{s}>2\rho_{m}时,I_{s}^{d^{*}}<I_{s}^{n^{*}};\;当\rho_{s}<2\rho_{m}时,I_{s}^{d^{*}}>I_{s}^{n^{*}} \end{array}$$

Among them, $I_{m}^{n^{*}}=\frac{\rho_{m}\left(\eta \rho +\eta \alpha +\delta \beta \right)}{k_{m}\left(\rho +\alpha \right)}$, $I_{s}^{n^{*}}=\frac{\rho_{s}\left(\eta \rho +\eta \alpha +\delta \beta \right)}{k_{s}\left(\rho +\alpha \right)}$ are the optimal level of green innovation efforts of manufacturers and suppliers in NASH non-cooperative game, respectively.

Proposition 3 shows that cost sharing, as an effective incentive behavior of green innovation activities in food supply chain, has its boundary condition. This means that within a certain threshold range, this kind of subsidy has a positive incentive effect, and makes the whole supply chain green innovation activities in the “double driving” mode, and manufacturers and suppliers have strong green innovation enthusiasm. Once it is beyond the threshold range, the impact of cost sharing on the green innovation activities of food supply chain is greatly reduced. At this time, manufacturers will not provide cost sharing to suppliers. The green innovation activities of the whole supply chain are in the “single driving mode.” The green innovation efforts of manufacturers and suppliers are only affected by the change of consumer behavior characteristics from the market demand side, and the impact of cost sharing can be ignored temporarily.

**Corollary 2:** At that time $\frac{\rho_{s}}{\rho_{m}}>2$, the incentive effect of cost sharing on suppliers was low, but both manufacturers and suppliers with a high level of green innovation efforts. This shows that in the mature stage of green innovation activities, manufacturers and suppliers get higher product margin through green innovation activities in the food supply chain, and food supply chain members are more spontaneous to carry out green innovation activities, and gradually achieve the balance between economic performance and environmental performance.

**Proposition 4:** In the green innovation activities that manufacturers provide cost sharing to suppliers, the cost sharing ratio of manufacturers to suppliers is positively related to the marginal profit of manufacturers, and negatively related to the marginal profit of suppliers.

$$\begin{array}{l} \text{Proof}:\varphi =\frac{2\rho_{m}-\rho_{s}}{2\rho_{m}+\rho_{s}},\frac{\partial \varphi }{\partial \rho_{m}}=\frac{4\rho_{s}}{{\left(2\rho_{m}+\rho_{s}\right)}^{2}}>0,\\ \frac{\partial \varphi }{\partial \rho_{s}}=-\frac{4\rho_{m}}{{\left(2\rho_{m}+\rho_{s}\right)}^{2}}<0, \end{array}$$

$$\begin{array}{l} \frac{\partial^{2}\varphi }{\partial \rho_{m}^{2}}=-\frac{16\rho_{s}}{{\left(2\rho_{m}+\rho_{s}\right)}^{3}}<0,\frac{\partial^{2}\varphi }{\partial \rho_{s}^{2}}=\frac{8\rho_{m}}{{\left(2\rho_{m}+\rho_{s}\right)}^{3}}>0 \end{array}$$

Proposition 4 shows that in the whole life cycle of green innovation activities in food supply chain, cost sharing by manufacturers to suppliers is an effective measure to stimulate suppliers to participate in green innovation activities. In this process, manufacturers will constantly adjust the cost sharing proportion of suppliers according to the actual situation, so as to ensure the maximization of both sides' green innovation benefits and promote green innovation in food supply chain system Efficiency improvement. Therefore, the cost sharing ratio is actually in a dynamic change. In order to improve the cost sharing ratio of manufacturers to suppliers in green innovation, the marginal profit of manufacturers can be increased and the marginal profit of suppliers can be reduced.

### Application Analysis

The third part of this paper proves and analyzes the influence of green innovation cost sharing on food supply chain equilibrium results through theoretical analysis. In order to verify the correctness of the proposed model and proposition, and further study its influence on food supply chain and enterprise net income, this section conducts sensitivity analysis on the cost sharing coefficient of green innovation through Mathematica Software. Two principles are adopted in the assignment of initial value of variable: one is to refer to the practice of relevant literature (Ramanathan et al., 2018; Li et al., 2019); the other is to assign median value when initial value of variable can be given relatively randomly without losing generality. Table 2 shows the initial value assignment of variables.

**Table 2:** Variable assignment.

**α**	**β**	**ρ**	**a**	**b**	**p**	***k*_*m*_**	***k*_*s*_**	***r*_0_**	**δ**	**η**	**ρ_m_**				**ρ_s_**			
2	0.5	0.1	20	1	20	5	3	7	1	0.5	4	6	8	10	2	4	6	8

According to the calculation results of the previous model, the cost sharing coefficient is determined by the marginal profit of the manufacturer and the supplier. This section mainly discusses the impact of the change of cost sharing coefficient on the green innovation efforts of manufacturers and suppliers in the food supply chain and the present value of profits. In order to more directly reflect the relationship between variables and the significance of their interaction, the sensitivity analysis in this section gives relatively high values when assigning marginal profits to manufacturers and suppliers as much as possible, so as to more intuitively describe how they affect the cost sharing ratio of manufacturers to suppliers. It also further analyzes how different sharing ratios cause green innovation efforts of manufacturers and suppliers is, and shows the degree of change of the food supply chain members have which have a certain impact on the present value of profits. Other variables are conventional parameter settings, mainly considering the influence of consumer green preference and reference price effect on green innovation activities.

**Figure 1:**
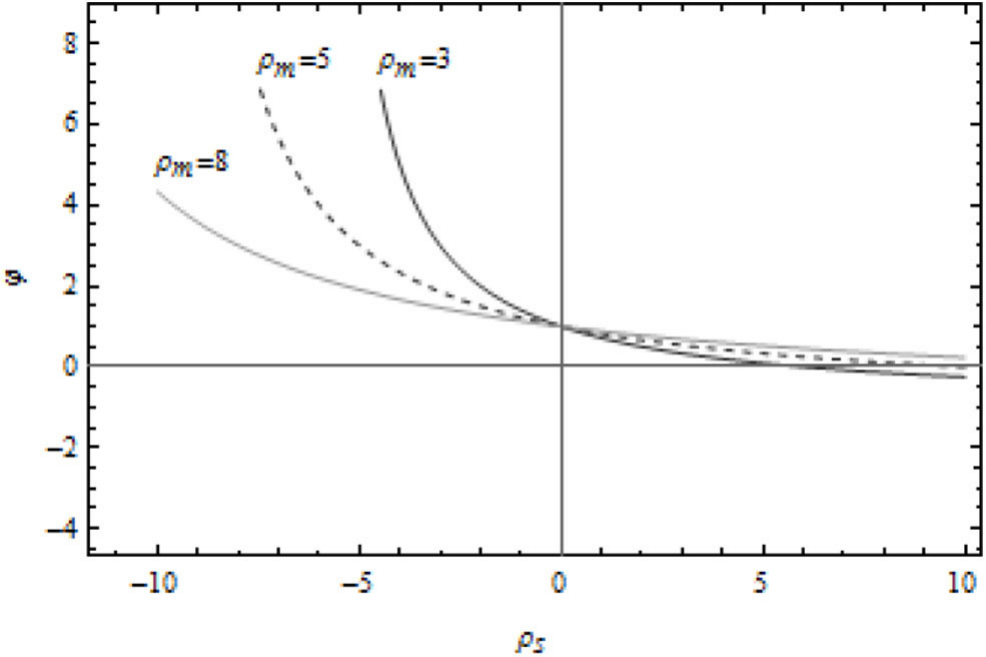
Relationship between cost sharing coefficient and marginal profit of suppliers.

From Figure 1, it can be seen that the cost sharing coefficient decreases with the increase of the supplier's marginal profit as a whole. The trend is moderate, and the final cost sharing coefficient is basically stable near “0.” In more details, when the cost sharing coefficient decreases with the increase of the manufacturer's marginal profit, the increase of the supplier's unit marginal profit has a greater impact on the cost sharing coefficient. When the cost sharing coefficient increases with manufacturer's marginal profit, the supplier's unit marginal profit has a relatively small impact on the cost sharing coefficient.

It can be found with further analysis that, first, the larger the marginal profit of the supplier unit, the larger the profit of the supplier through the green innovation activities, the larger the profit space of the green products, and the smaller the incentive effect of cost sharing. The supplier will spontaneously carry out the green innovation activities, which is mainly stimulated by the green consumption at the market demand end. Second, the marginal profit of the supplier unit is In the case of negative value, the greater the manufacturer's marginal profit is, the more likely it is to share the cost of green innovation activities for suppliers, or even provide them with green information resource sharing, so as to ensure the stable supply of goods by suppliers and form a long-term strategic cooperation relationship. Thirdly, in the case of positive marginal profit of suppliers, the stronger the supplier's profitability is, and vice versa. With the continuous growth of green economy, green manufacturing has gradually evolved into an endogenous variable of enterprises. Enterprises in the food supply chain carry out green innovation activities with upstream and downstream enterprises to form core competitiveness, while the “capital sharing” of the cost of green innovation in the early stage will eventually feedback large manufacturers and enterprises, so as to keep leading in the green driven economic environment First place.

**Figure 2:**
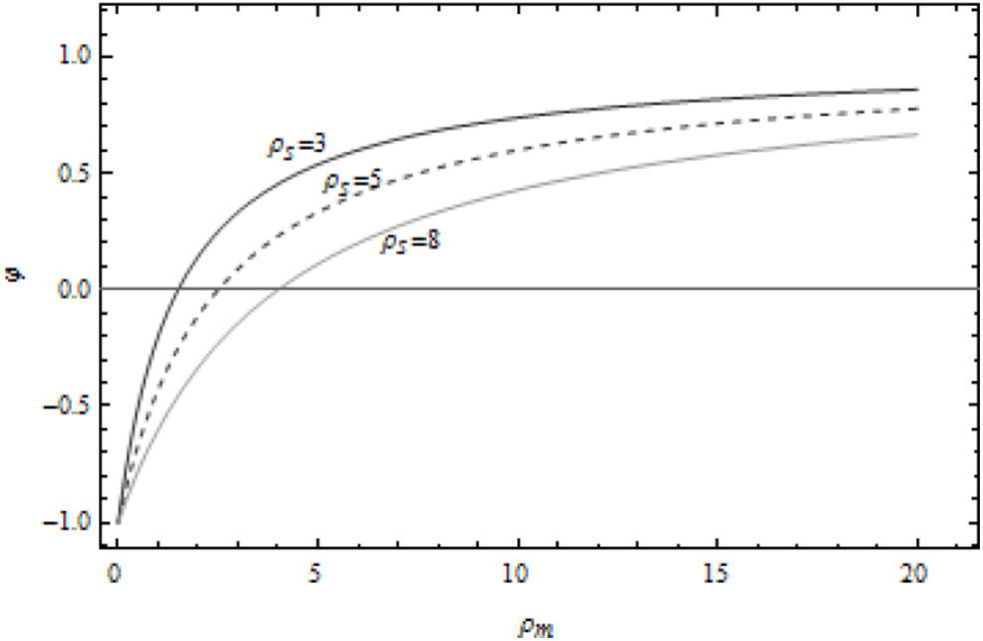
Relationship between cost sharing coefficient and manufacturer's marginal profit.

It can be seen from Figure 2 that when the marginal profit of the supplier is fixed, the cost sharing coefficient increases with the increase of the marginal profit of the manufacturer. When the marginal profit of the manufacturer is fixed, the cost sharing coefficient decreases with the increase of the marginal profit of the supplier. In more detail, under the same conditions, the cost sharing coefficient of the supplier ρ_*s*_ = 3 is the largest, and the marginal profit of the supplier ρ_*s*_ = 5 is the largest. The cost sharing coefficient is in the middle, and the supplier's marginal profit ρ_*s*_ = 8 is the smallest. This shows that the cost sharing of manufacturers to suppliers is closely related to their own economic strength and marginal profit of suppliers. In essence, the green innovation activity of food supply chain is a “game” of manufacturers and suppliers' green innovation efforts. When both sides of the game are rational people, manufacturers will adjust the cost sharing ratio of suppliers according to the actual situation to achieve win-win.

**Figure 3:**
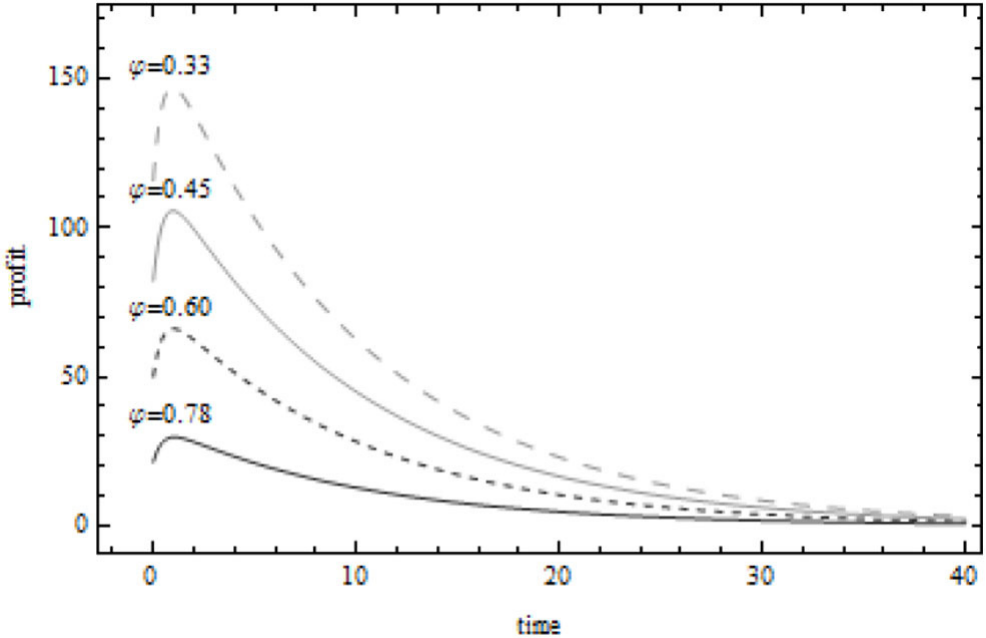
Relationship between present value of supplier profit and cost sharing coefficient.

When the manufacturer's marginal profit ρ_*m*_ = 8, Figure 3 describes the change of the present value of the supplier's profit when the value of the supplier's marginal profit is ρ_*s*_ = 2, ρ_*s*_ = 4, ρ_*s*_ = 6, ρ_*s*_ = 8, respectively. The supplier's profit decreases with the increase of time. After obtaining high economic benefits in the early stage of green innovation activities, the supplier's economic benefits gradually decrease, which is mainly affected by the technical difficulty and uncertain benefits of green innovation activities, as well as the subsequent more enterprises joining in green innovation activities, forming a fierce competition relationship with each other. At the same time, enterprises will pay more attention to the environment in the future environmental performance, not economic efficiency. Further observation shows that when the marginal profit of the supplier is certain and greater than the marginal profit of the supplier, with the increase of the marginal profit of the supplier, the cost sharing proportion of the manufacturer to the supplier decreases, while the marginal profit of the supplier increases continuously, which further shows that the cost sharing of the manufacturer to the supplier is not always effective, and there is a threshold value within the threshold range. Cost sharing can become the “push hand” of green innovation activities in food supply chain and realize the green innovation incentive of manufacturers to suppliers.

**Figure 4:**
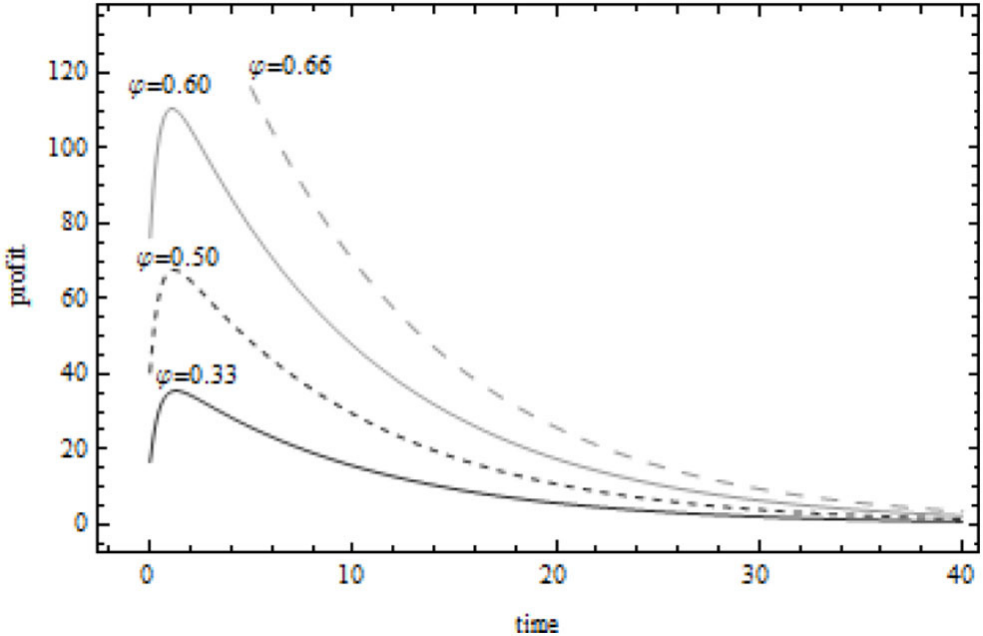
Relationship between present value of manufacturer's profit and cost sharing coefficient.

When the supplier's marginal profit ρ_*s*_ = 4, Figure 4 describes the change of the present value of the manufacturer's profit when the value of the manufacturer's marginal profit is ρ_*m*_ = 4, ρ_*m*_ = 6, ρ_*m*_ = 8, ρ_*m*_ = 10, respectively. When the marginal profit of the supplier is certain and less than the marginal profit of the manufacturer, the greater the marginal profit of the manufacturer, the greater the cost sharing proportion of the manufacturer to the supplier. Under the condition that the present value of the profit obtained by the manufacturer through green innovation activities is more similar, the present value of the manufacturer's unit profit increases first, then less and then increases with the increase of its own marginal profit. According to Figures 3, 4, when δ = 0.6(ρ_*m*_ = 8, ρ_*s*_ = 4), the present value of the manufacturer's profit is 140 units, and the present value of the supplier's profit is 65 units. This shows that both manufacturers and suppliers can obtain considerable benefits from green innovation activities in the food supply chain. The cost sharing contract, as the “promoter” of the cooperation between the upstream and downstream enterprises in the food supply chain, can indeed stimulate the green innovation activities of both sides in the food supply chain. More importantly, through the form of cost sharing, manufacturers and suppliers establish a trust mechanism to ensure their own long-term stable supply, reduce the uncertainty in green innovation activities, and realize Pareto improvement of food supply chain system profits.

## Conclusion

In this paper, a food supply chain green innovation model based on Stackelberg game is established to describe the situation that manufacturers encourage suppliers' green innovation efforts through cost sharing contract. On this basis, the changes of manufacturers' and suppliers' optimal green innovation efforts, optimal profit level and subsidy effectiveness caused by the change of cost sharing coefficient are discussed. The following results is concluded.

First, in the Stackelberg game between manufacturer and supplier, the non-linear change of cost sharing will affect the green innovation decision of food supply chain, and there is an optimal sharing threshold $\frac{\rho_{s}}{\rho_{m}}=2$. At that time $\frac{\rho_{s}}{\rho_{m}}\leq -2$, there will be supply interruption. Manufacturers need to stimulate suppliers to make green innovation efforts through cost sharing. At this time, the cost sharing ratio is higher than normal.

Second, at that time $-2<\frac{\rho_{s}}{\rho_{m}}\leq 0$, the optimal level of green innovation efforts of manufacturers and suppliers is positive. At this time, the whole supply chain green innovation activities were in efficient operation and could achieve considerable economic benefits. Food supply chain members are in the “double driving” mode, that is, sustainability is encouraged by the green preference of consumers from the market demand side, the reference price effect and cost sharing. Among them, the market demand side incentives account for the main part.

Third, at that time $0<\frac{\rho_{s}}{\rho_{m}}\leq 2$, cost sharing had an obvious incentive effect on suppliers' green innovation efforts, and the incentive effect of cost sharing was better than that of consumer's green preference and reference price effect at the market demand end. The cost sharing ratio of manufacturers to suppliers was positively related to manufacturers' marginal profits, and negatively related to suppliers' marginal profits.

From the overall content of the paper, the green innovation efforts of manufacturers and suppliers will be stimulated by the situation of consumers in the market. At the same time, the degree of green innovation efforts of suppliers will also be stimulated by the cost sharing of manufacturers. In terms of suppliers, the possible incentives are twofold, which can bring strategic suggestions for enterprises. First, in the early stage of the market, due to the high risk and uncertainty of green innovation activities in the food supply chain, the market scale of green products has not yet formed, which is prone to the problem of insufficient incentive to suppliers, and further leads to supply interruption. Therefore, large manufacturers need to pay close attention to the economic situation of upstream and downstream enterprises, establish good strategic cooperation relationship, ensure the sustainable development of green innovation activities in the food supply chain, create products with low energy consumption and high green degree, and establish green barriers for enterprises. Second, cost sharing is a powerful method for incentive of green innovation activities in food supply chain. Considering the advantages of manufacturers' rights, information and the need to cultivate strategic partners, large manufacturers can greatly encourage suppliers' green innovation efforts through cost sharing. And the cost sharing ratio can be determined according to the cooperation between the two sides, so as to achieve effective incentive and Pareto improvement of food supply chain profits. Thirdly, the green innovation activities of food supply chain are influenced by the green preference of consumers from the demand side of external market and the reference price effect, and the incentive of internal cost sharing means, and the incentive of cost sharing depends on the manufacturer's response to the demand side of external market. This requires enterprises not only to coordinate internal resources, but also to accurately grasp consumer preferences and make rapid product line decisions under the background of green consumption and service becoming the mainstream trend.

There are still some limitations in this study, which need to be extended in the future research. Firstly, the object of this research is constrained to a two-level food supply chain system composed of a manufacturer and a supplier, which can be introduced to the competitive environment of suppliers. Secondly, the cost sharing as an incentive means in this paper, but manufacturers can achieve effective incentives for suppliers through a variety of combinations. Thirdly, The simulation study of this paper only represents a purely rationale account of the phenomenon explored and cannot sufficiently interpret the practice of cooperation innovation in food industry supply chain. Therefore, further research can explore the feasibility of cooperation models bedsides cost sharing, such as wholesale price premium contract in supply chain green innovation activities.

## Data Availability Statement

All datasets presented in this study are included in the article/supplementary material.

## Author Contributions

JH and YL wrote the first draft of the original manuscript. YL and XF analyzed the major model and simulation. C-HL and C-HC conceptualized, reviewed, and edited the paper. All authors contributed to the article and approved the submitted version.

## Conflict of Interest

The authors declare that the research was conducted in the absence of any commercial or financial relationships that could be construed as a potential conflict of interest. The reviewer C-HL declared a shared affiliation, with no collaboration, with one of the authors, C-HL and C-HC, to the handling editor at time of review.
